# Factors Determining Disease Duration in Alzheimer's Disease: A Postmortem Study of 103 Cases Using the Kaplan-Meier Estimator and Cox Regression

**DOI:** 10.1155/2014/623487

**Published:** 2014-01-22

**Authors:** R. A. Armstrong

**Affiliations:** Vision Sciences, Aston University, Birmingham B4 7ET, UK

## Abstract

Factors associated with duration of dementia in a consecutive series of 103 Alzheimer's disease (AD) cases were studied using the Kaplan-Meier estimator and Cox regression analysis (proportional hazard model). Mean disease duration was 7.1 years (range: 6 weeks–30 years, standard deviation = 5.18); 25% of cases died within four years, 50% within 6.9 years, and 75% within 10 years. Familial AD cases (FAD) had a longer duration than sporadic cases (SAD), especially cases linked to *presenilin* (*PSEN*) genes. No significant differences in duration were associated with age, sex, or *apolipoprotein E* (*Apo E*) genotype. Duration was reduced in cases with arterial hypertension. Cox regression analysis suggested longer duration was associated with an earlier disease onset and increased senile plaque (SP) and neurofibrillary tangle (NFT) pathology in the orbital gyrus (OrG), CA1 sector of the hippocampus, and nucleus basalis of Meynert (NBM). The data suggest shorter disease duration in SAD and in cases with hypertensive comorbidity. In addition, degree of neuropathology did not influence survival, but spread of SP/NFT pathology into the frontal lobe, hippocampus, and basal forebrain was associated with longer disease duration.

## 1. Introduction

Studies of the life expectancy of patients with Alzheimer's disease (AD) are important in calculating prevalence rates while identifying factors that influence survival is useful both in counseling patients and their families and in public health planning [1–3]. Many published studies, however, suggest that survival rates vary considerably in AD and depend on numerous factors [3]. Hence, survival may depend on age at diagnosis, sex, disease subtype, and severity of progression of the disease.

AD is a heterogeneous disease [4–6] and survival may depend on subtype [7]. First, AD consists of sporadic (SAD) and familial forms (FAD), most cases of the latter being associated with mutations of either the* amyloid precursor protein* (*APP*) gene on chromosome 21 [8, 9] or the *presenilin* genes *PSEN1* [10] and *PSEN2* [11] on chromosomes 14 and 1, respectively. In addition, allelic variation in the *apolipoprotein E* (*Apo E*) gene on chromosome 19 has been identified as an important risk factor, especially in late-onset AD individuals having 2-3 times the frequency of allele *ε*4 compared with nondemented elderly controls [12]. Second, more complex forms of AD have been described, for example, AD in combination with parkinsonism [13] or dementia with Lewy bodies (DLB/AD) [14], with significant degeneration of white matter [15] or with capillary amyloid angiopathy (CAA). Hence, the presence of one or more comorbidities may significantly affect survival of AD patients. Third, studies suggest at least two distinct types of disease progression in AD, namely, a rapidly progressive subtype with a median survival time of 10 months and a subtype characterized by a much slower progression [16–18].

The objective of the present study was to investigate the relationship between genetics, demography, neuropathology, comorbidity, and disease duration in 103 AD cases. The Kaplan-Meier estimator and Cox regression analysis (proportional hazard model) were used to study the survival function of the AD patients and to test specific hypotheses [19–21].

## 2. Materials and Methods

### 2.1. Cases

The study population comprised a series of 103 consecutive cases of AD diagnosed at the Department of Neuropathology, University of Washington, Seattle, USA (Table 1) [6]. Informed consent was given for the removal of all brain tissue according to the Declaration of Helsinki. AD cases all fulfilled “National Institute of Neurological and Communicative Disorders and Stroke and the Alzheimer Disease and Related Disorders Association” (NINCDS/ADRDA) criteria for probable AD [22] and were neuropathologically verified using “Consortium to Establish a Registry of Alzheimer Disease” (CERAD) criteria [23] and National Institute on Aging and Reagan Institute criteria [24]. All cases conformed to stage V or VI of the Braak staging system [25, 26]. The family history of all cases was examined and those with at least one or more first-degree relatives affected were regarded as FAD. Disease duration was measured from the onset of dementia, which was determined by clinical assessment, and defined as cognitive dysfunction sufficiently severe to impair activities of daily living. Of the 103 cases, 19/103 (18%) were early-onset (≤65 yrs) FAD, 12/103 (11.6%) were late-onset (>65 yrs) FAD, 22/103 (21%) were early-onset SAD, and 50/103 (49%) were late-onset SAD. Nine of the early-onset FAD cases were linked to *PSEN* genes, four cases to *PSEN1*, and five cases to *PSEN2*. *Apo E* genotype was determined for a subset of 40/103 of the cases with the following distribution of genotypes: *ε*2/3 (*n* = 1), *ε*3/3 (*n* = 12), *ε*2/4 (*n* = 3), *ε*3/4 (*n* = 18), and *ε*4/4 (*n* = 6).

### 2.2. Neuropathology

Total brain weight was measured at postmortem. In addition, the abundance of senile plaques (SP) and neurofibrillary tangles (NFT), the signature pathological lesions of AD [23, 27], was measured in 22 cortical and subcortical brain areas from each case (Table 2). The quantitative assessment of SP and NFT was based on examination of tissue sections stained by silver impregnation methods (Holmes, Bielschowsky) which stain mature “neuritic” and “classic” SP and NFT [6]. Preamyloid or diffuse plaques are not consistently labeled by these methods. In each region, 10, 50 × 50 *μ*m sample fields were located at random. The abundance of SP and NFT within each field was assessed using a semiquantitative scale [28]: 0 = none, 1 = mild, 2 = moderate, and 3 = severe. The abundance scores of the SP and NFT were averaged to give a single abundance score for each brain area and rounded up to the nearest whole number. The mean abundance score of the SP/NFT derived from all brain areas studied was used as a measure of the overall severity of the pathology in each case.

### 2.3. Comorbidity

Comorbidity data [4] were obtained from postmortem records and divided into two groups: (1) neurological comorbidity, for example, associated parkinsonism, amyotrophic lateral sclerosis (ALS), Pick's disease (PiD), Creutzfeldt-Jakob disease (CJD), stroke, infarct, aneurysm, hydrocephalus, motor neuron disease (MND), or tumor, and (2) nonneurological comorbidity, for example, cardiovascular disease (CVD), arterial hypertension, respiratory disease, malignant disease, diabetes, and anemia. A number of aspects of comorbidity were tested against survival of AD patients: (1) the presence or absence of a neurological or nonneurological comorbidity, (2) the frequency of neurological or nonneurological comorbidities recorded for each case, and (3) the individual types of neurological and nonneurological comorbidity which were present in at least six cases.

### 2.4. Data Analysis

The Kaplan-Meier estimator (“product limit estimator”) was used to study the overall pattern of survival among the 103 cases and is the fraction of patients which survive for a certain period after onset of dementia. In typical applications, the data can be grouped into subtypes and the effect of the grouping factor on disease duration estimated. Where two groups were present, for example, FAD/SAD, male/female, or presence/absence of comorbidity, duration was compared using the Gehan-Wilcoxon two-sample test. With more than two groups, duration was compared using the chi-square (*χ* ^2^) test. The Cox proportional hazard model allows the relationship between disease duration and continuously measured variables to be tested and was applied to (1) demographic variables such as patient age and disease onset, genetic factors such as *Apo E* score (the sum of the two alleles), and the frequency of one or more neurological and nonneurological comorbidities, (2) measures of overall neuropathology such as mean severity score and the number of brain areas affected by SP/NFT, and (3) abundance scores of SP and NFT in each of the 22 brain areas investigated. The effect of disease onset may be biased by the presence of cases linked to *PSEN* mutations, and hence, the effect of this variable was also assessed with the *PSEN* mutation cases omitted. Statistical significance was based on “*t*” and the “Wald” statistic.

## 3. Results

**Figure 1:**
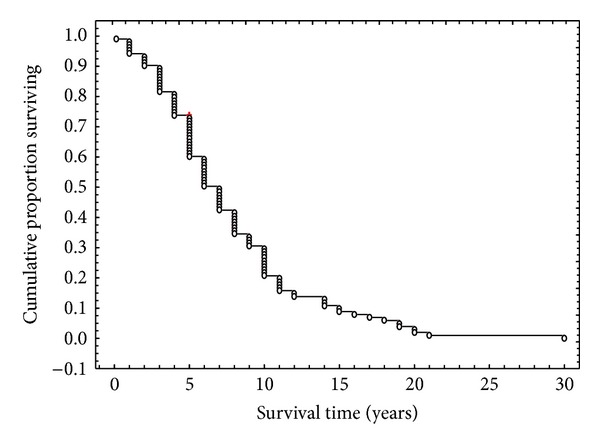
Kaplan-Meier survival function of all 103 Alzheimer's disease (AD) patients.

Mean disease duration of the 103 AD cases was 7.1 years (range: 6 weeks–30 years, standard deviation = 5.18). The survival function for all 103 cases studied is shown in Figure 1. The data indicate that 25% of cases died within four years, 50% within 6.9 years, and 75% within 10 years after onset.

**Figure 2:**
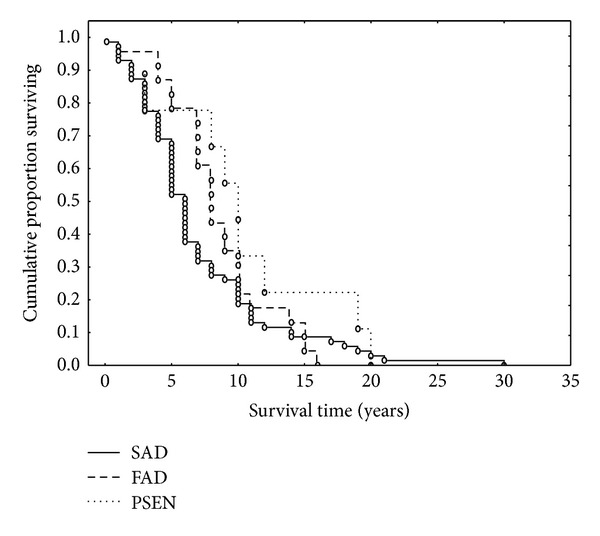
Kaplan-Meier survival function of the data grouped into familial Alzheimer's disease (FAD), sporadic Alzheimer's disease (SAD), and familial cases linked to *presenilin* (*PSEN*) mutations (comparison between groups: *χ* ^2^ = 7.13, *P* < 0.01).

**Figure 3:**
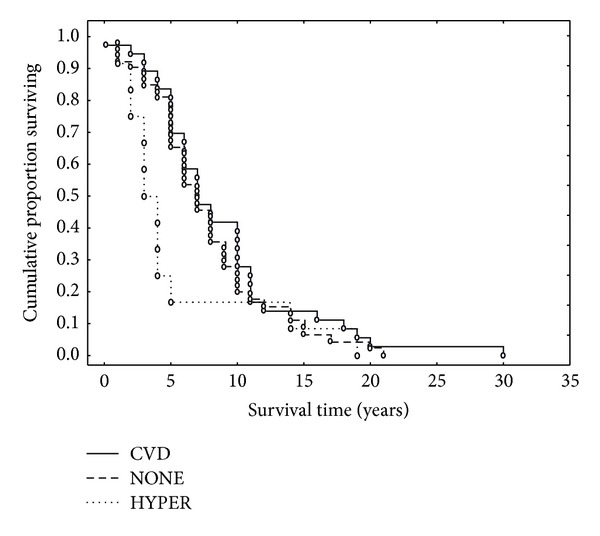
Kaplan-Meier survival function of the data grouped into those Alzheimer's disease (AD) patients with no associated signs of cardiovascular disease (NONE), those with the signs of cardiovascular disease (CVD), and those with arterial hypertension (HYPER) (comparison between groups: *χ* ^2^ = 8.50, *P* < 0.05).

Gehan-Wilcoxon test suggested a significant difference in disease duration between SAD and all FAD cases (G-W = 2.51, *P* < 0.05). When cases were separated into SAD, cases linked to *PSEN* mutations, and all remaining FAD (Figure 2), the *PSEN* cases, on average, exhibited the greatest disease duration (*χ* ^2^ = 7.13, *P* < 0.01). There were no significant differences in duration when the data were grouped according to sex (G-W = 0.58, *P* > 0.05), the presence/absence of neurological comorbidity (G-W = 0.51, *P* > 0.05), or individual types of neurological comorbidity (*χ* ^2^ = 12.15, *P* > 0.05). Similarly, the presence of a nonneurological comorbidity did not affect overall duration (G-W = 0.78, *P* > 0.05). Nevertheless, when data were grouped according to the presence of CVD, arterial hypertension, or neither of these, duration was reduced in the hypertensive group (Figure 3) (*χ* ^2^ = 8.50, *P* < 0.05).

The results of the Cox regression analysis for demographic variables, *Apo E* genotype, and the neuropathological variables are shown in Table 3. The data suggest that (1) patient age had no significant effect on duration, (2) disease onset was significantly associated with duration, early-onset cases exhibiting better survival (*t* = 4.12, Wald statistic = 16.98, and *P* < 0.01), (3) *Apo E* score and brain weight were unrelated to duration, and (3) neither total number of areas affected by SP/NFT nor overall severity of pathology was significantly related to duration. The effect of disease onset was similar regardless of whether the PSEN mutation cases were included in the analysis (*t* = 4.75, *P* < 0.001).

The results of the Cox regression analysis of the SP/NFT scores from each brain area studied are shown in Table 4. Of the 22 areas examined, increasing disease duration was associated with increased abundance scores of SP/NFT in three specific areas, namely, the orbital gyrus (OrG) (*t* = 2.03, Wald statistic = 4.12, and *P* < 0.05), CA1 sector of the hippocampus (*t* = 2.04, Wald statistic = 4.18, and *P* < 0.05), and nucleus basalis of Meynert (NBM) (*t* = 2.09, Wald statistic = 4.38, and *P* < 0.05).

## 4. Discussion

Mean disease duration of the 103 AD cases studied was 7.1 years slightly higher than the 5.2 years and 6.5 years recorded by Doody et al. [29] and Feldman et al. [30], respectively. The data also contrast with those reported for a specific group of AD cases, namely, those with vascular disease comorbidity, in which mean survival was less than five years [30], and semantic dementia (SD), in which 50% of patients survived more than 12.8 years [20]. In addition, although controversial, recent evidence supports the presence of two distinct subtypes of AD progression [17], cases having either a very short (median survival 10 months) or a significantly longer disease duration. AD cases with very short durations were evident in the present sample, 10 cases surviving two years or less. A multiple discriminant analysis comparing these 10 cases with the remainder suggested that short duration was not related to age, onset, brain weight, neuropathology, or comorbidity. The rapidly progressive form of AD has been linked to increased levels of education and to be associated with a more global cognitive impairment [31]. In addition, a strong correlation between AD survival and rate of cognitive decline was reported by Hui et al. [32]. By contrast, Bruandet et al. [33] found that highly educated individuals with AD had a faster rate of cognitive decline but did not have reduced survival times. A limitation of the study is that no accurate data were available on the education level of sufficient cases to test this hypothesis. Alternatively, timing to onset of clinical diagnosis may be related to undiagnosed pathology such as vascular disease or spongiform change.

No significant differences in duration were observed between males and females. This result contrasts with some earlier studies which often show poorer survival in males [1, 29, 34, 35]. However, the data suggested longer disease duration in FAD than SAD, especially in cases linked to *PSEN* mutations. Variation in survival rates between FAD and SAD could result from differences in neuropathology. Hence, cases linked to *PSEN1* have greater numbers of SP and NFT compared with cases of SAD [36] but this increased pathology would be expected to shorten survival rates. In addition, many studies suggest that FAD and SAD have essentially the same pathology [37–39]. The most likely explanation for the longer duration of the FAD cases is either earlier disease onset or reduced survival in SAD as a consequence of comorbidity.

Various types of neurological comorbidity were not associated with shorter durations, in contrast with the study of Feldman et al. [30], which showed reduced survival in cases with associated vascular dementia. Similarly, the presence/absence of nonneurological comorbidity did not reduce survival. In previous studies, the presence of at least one disease complication decreased lifespan in AD [40] and the presence of comorbidity and functional disability was an important predictor of survival [21]. Bowen et al. [1] found a strong association between decreased survival in AD and vascular disease which is regarded as a significant determinant of progression to dementia. A limitation of the present study is that accurate quantitative data on CVD load, for example, lacunar infarcts, microinfarcts, and atherosclerosis of large vessels, was not available. However, in the present study, the presence/absence of CVD was not associated with disease duration. Nevertheless, there was evidence of reduced disease duration in those AD cases with arterial hypertension when compared with those with CVD or neither of these conditions. Hypertension is a strong risk factor for stroke, heart disease, aneurysm, and chronic kidney disease but the sample of cases was too small to test whether hypertensive individuals had increased incidence of these pathologies. A strong correlation between the presence of atherosclerosis and mortality in AD [41] and between survival and severe arterial hypertension, measured at the beginning of the study [19], has been demonstrated in AD. By contrast, Weiner and Risser [42] found no effect of CVD or hypertension on AD survival. In addition, no association between survival and CVD was found in a cohort of Down's syndrome (DS) patients [43], who frequently develop AD-type pathology [44–46].

No association was observed between disease duration and *Apo E* genotype of the patient. This is surprising as the presence of allele *ε*4 often accelerates the development of AD pathology [47] and hence is associated with an earlier disease onset [48]. *Apo E* genotype was available for only 40 of the cases studied, which was too small a sample size to determine the true effect of this variable. In the rapidly progressive form of AD, however, no association with *Apo E* allele *ε*4 and survival was reported [18]. By contrast, Tilvis et al. [49] found that the presence of *Apo E *ε**4 allele was associated with impaired cognitive function and clinical dementia and hence could be associated with reduced survival.

Whether the degree of brain atrophy and weight significantly change over the course of AD has been controversial. There are significant limitations in studying this complex variable as many factors may influence brain weight such as height of the subject and the degree of osteoporosis. In one study, poorer survival was associated with lower gray matter volume and smaller volume reductions in brain predicted better survival [50]. In the present study, no relationship between brain weight and disease duration was demonstrated. In addition, neither total number of areas affected by SP/NFT pathology nor an overall measure of severity of the pathology was associated with disease duration suggesting neuropathology does not directly affect survival. Nevertheless, data suggest an association between SP/NFT pathology in the OrG, sector CA1 of the hippocampus, and NBM and longer disease durations consistent with spread of the pathology into these areas later in the disease. Alternatively, correlation of longer duration to pathology may result from regions being the earliest affected and therefore exposed to accumulating pathology over periods of time. Several studies suggest that the pathology of AD may spread through the brain via anatomical connections from an origin in the temporal lobe to the cortical association areas and hippocampus and then to the primary sensory areas [51–55]. In addition, pathogenic proteins, such as tau, *α*-synuclein, and *β*-amyloid (A*β*), can be secreted from cells, enter other cells, and seed small intracellular aggregates within these cells [56, 57] and therefore could spread through the brain.

In conclusion, the data suggest disease duration of AD patients is reduced in SAD and especially in cases with associated arterial hypertension. By contrast, sex, neurological comorbidity, brain weight, *Apo E* genotype, and neuropathology had little effect on survival. Longer duration cases, however, were associated with spread of SP/NFT pathology into the frontal lobe, hippocampus, and basal forebrain.

## Figures and Tables

**Table 1:** Demographic details of the 103 cases used in the study (*N*: number of cases; FAD: familial Alzheimer's disease; SAD: sporadic Alzheimer's disease). Data for age at death, duration, and disease onset are means with range and standard deviations in parentheses.

Patient group	*N*	Age at death (years)	Duration (years)	Onset (years)
Early-onset FAD	19	61.9 (46–74, 10.8)	11.1 (3–20, 6.5)	50.7 (38–59, 6.5)
Early-onset SAD	22	70.4 (57–88, 11.1)	16.0 (6–30, 9.3)	54.4 (49–58, 4.2)
Late-onset FAD	12	77.4 (70–85, 5.1)	7.0 (1–15, 4.6)	70.4 (61–84, 7.6)
Late-onset SAD	50	80.1 (70–98, 6.6)	6.8 (1–21, 4.5)	73.5 (62–93, 7.2)

**Table 2:** Brain regions and areas studied.

Brain region	Area studied	Abbreviation
Frontal	Superior frontal gyrus	SFG
	Orbital gyrus	OrG
	Gyrus rectus	GyR
	Cingulate gyrus	CgG

Perisylvian	Insula/claustrum	In/Cl

Temporal	Superior temporal gyrus	STG
	Parahippocampal gyrus	PHG
	Hippocampus, CA1	CA1
	Dentate gyrus	DG
	Amygdala	Am

Parietal	Superior parietal lobe	SPL

Occipital	Visual cortex (B17/B18)	OC

Subcortical	Thalamus	Th
	Lateral geniculate nucleus	LGN
	Basal ganglia	BG
	Substantia nigra	SN
	Nucleus basalis of Meynert	NBM
	Ventral tegmentum	VT
	Raphe nucleus	RaN
	Mamillary bodies	MB
	Hypothalamus	HyP
	Cerebellum	CB

**Table 3:** Results of the Cox proportional hazard model analysis for demographic, *apolipoprotein E* (*Apo E*) score, overall severity of neuropathology, and comorbidity (the frequency of neurological and nonneurological comorbidities present in each case).

Variable	*β*	SE	*t*	Wald	*P*
Patient age	−0.005	0.01	0.47	0.99	0.64
Disease onset (all cases)	0.049	0.01	4.12	16.98	0.008**
Disease onset (minus *PSEN* cases)	0.055	0.012	4.75	22.61	<0.001
Brain weight	0.001	0.0009	0.62	0.38	0.54
*Apo E* score	0.079	0.27	0.29	0.08	0.77
Number of areas affected	−0.002	0.056	0.04	0.001	0.97
Overall severity score	−0.011	0.058	0.19	0.03	0.85
Neurological comorbidity	0.131	0.136	0.96	0.93	0.33
Nonneurological comorbidity	0.032	0.081	0.40	0.16	0.70

*β*: regression coefficient; SE: standard error; *P*: probability; **P < 0.01.

**Table 4:** Results of the Cox proportional hazard model analysis for abundance scores of senile plaques (SP) and neurofibrillary tangles (NFT) in each brain region.

Area	*β*	SE	*t*	Wald	*P*
Superior frontal gyrus	−0.00329	0.01	0.31	0.098	0.75
Cingulate gyrus	−0.00998	0.02	0.44	0.99	0.66
Orbital gyrus	0.02039	0.01	2.03	4.12	0.04*
Gyrus rectus	−0.1717	0.03	0.62	0.98	0.53
Amygdala	0.01040	0.01	1.31	1.01	0.19
Dentate gyrus	0.00544	0.03	0.19	1.00	0.85
Insula	−0.00252	0.01	0.26	0.07	0.79
Claustrum	0.03414	0.02	1.71	2.92	0.09
Parahippocampal gyrus	−0.00190	0.01	0.17	0.03	0.86
Hippocampus CA1	0.03575	0.02	2.04	4.18	0.04*
Superior temporal gyrus	0.00699	0.01	0.74	0.55	0.46
Ventral tegmentum	0.00470	0.02	0.26	1.00	0.80
Raphe nucleus	−0.02172	0.02	1.11	1.24	0.27
Substantia nigra	0.02086	0.04	0.47	0.22	0.64
Thalamus	−0.02530	0.02	1.58	2.51	0.11
Visual cortex (B17/B18)	−0.00912	0.01	0.98	0.95	0.33
Superior parietal	−0.01378	0.01	1.42	2.02	0.15
Nucleus basalis of Meynert	0.03978	0.02	2.09	4.38	0.04*
Mamillary bodies	0.01486	0.02	0.78	0.59	0.44
Hypothalamus	0.01424	0.02	063	0.40	0.53
Basal ganglia	−0.02667	0.02	1.17	1.37	0.24
Cerebellar cortex	−0.0297	0.02	1.41	1.97	0.16

*β*: regression coefficient; SE: standard error; *P*: probability; *P < 0.05.
